# Supplementary material for: Causal Relationship between Aging and Anorexia Nervosa: A White-Matter-Microstructure-Mediated Mendelian Randomization Analysis
Source: Biomedicines. 2024 Aug 16;12(8):1874. doi: 10.3390/biomedicines12081874 (PMC11351342; doi:10.3390/biomedicines12081874)
Supplement: Supplementary file 1 [file biomedicines-12-01874-s001.zip › Supplementary Figure/Figure S8/Leaveoneout plot of MR analysis for TL to ubm-b-1908.pdf]

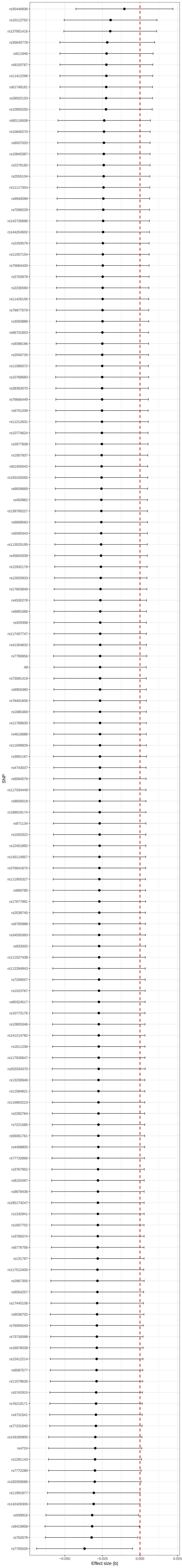

rs35446936
rs10112752
rs137901416
rs35640778
rs611646
rs8105767
rs11412296
rs61748181
rs28502153
rs10905255
rs80116508
rs10840270
rs6007020
rs10845387
rs2276182
rs2555104
rs11117354
rs9940099
rs7099229
rs142730696
rs144204502
rs2293579
rs11557154
rs75664430
rs2763979
rs2230590
rs11426156
rs79977579
rs3093888
rs66731853
rs9398196
rs2056726
rs11085072
rs10768683
rs28363070
rs76666449
rs6751209
rs11212631
rs10774624
rs2977608
rs1957937
rs61405042
rs150150565
rs6659669
rs450962
rs139795227
rs6669563
rs6590343
rs113525195
rs45604339
rs12932179
rs12925933
rs17803849
rs4530278
rs6881568
rs429358
rs117407747
rs41304832
rs7790856
All
rs73581419
rs9955360
rs78491606
rs1985369
rs11769630
rs4616688
rs11699829
rs3891167
rs4743037
rs6584579
rs117034449
rs9600019
rs188918174
rs871134
rs1003322
rs12451892
rs145114957
rs376641875
rs111950327
rs869785
rs17677991
rs2538745
rs6790988
rs34550383
rs932002
rs111527438
rs112394943
rs7209057
rs1023767
rs80324517
rs10773176
rs10805346
rs141214782
rs1611236
rs117630647
rs202034370
rs13230646
rs11584821
rs116863223
rs2282764
rs7221585
rs56061761
rs4498805
rs77732866
rs3767952
rs8102497
rs9878436
rs185174247
rs1332941
rs1907702
rs3785074
rs6776756
rs131797
rs117512405
rs2967355
rs6054257
rs17445108
rs6536702
rs76065543
rs73730598
rs16978028
rs12412214
rs6587577
rs11579626
rs5742915
rs76219171
rs4731541
rs77231040
rs143190905
rs4724
rs1291143
rs7772289
rs182059586
rs11991877
rs142426306
rs939916
rs9419958
rs762679
rs7705526
SNP
−0.050
−0.025
0.000
0.025
Effect size (b)
